# AI-assisted diagnostic approach for the influenza-like illness in children: decision support system for patients and clinicians

**DOI:** 10.1007/s13534-024-00450-8

**Published:** 2024-12-30

**Authors:** Youngro Lee, Jongmo Seo, Yun-Kyung Kim

**Affiliations:** 1 Department of Electrical and Computer Engineering, Seoul National University, Seoul, South Korea; 2 Biomedical Research Institute, Seoul National University Hospital, Seoul, South Korea; 3 Department of Pediatrics, College of Medicine, Korea University, Seoul, South Korea

**Keywords:** Influenza-like illness, Decision support system, Machine learning, Shapley additive explanations

## Abstract

Influenza-like illnesses (ILI), such as influenza and RSV, pose significant global health burdens, especially in febrile children under 6 years old. Differentiating these from bacterial infections based solely on clinical symptoms is challenging. While PCR tests are reliable, they are costly and time-consuming. An effective predictive tool would help doctors prioritize tests and guide parents on seeking emergency care for their febrile children. We collected data from 2,559 children who visited the hospital for ILI inspections. We developed XGBoost models, comparing nine different machine learning algorithms. Our AI-assisted diagnostic pipeline consists of two stages: Decision Support System for patients (DSS-P): An in-house model using sex, age, symptoms, and medical history to decide on hospital visits. Decision Support System for clinicians (DSS-C): An in-hospital model incorporating breath sound types and Chest X-ray results to determine the necessity of clinical tests. We tested various experimental settings, including the addition of RAT-tested samples and the combined consideration of influenza and RSV. The performance for influenza achieved an Area Under the Curve of 0.749 and 0.776, while RSV achieved 0.907 and 0.924 in DSS-P and DSS-C, respectively. We identified biomarkers, noting that most biomarkers had opposite effects for influenza and RSV. This study developed predictive models for influenza and RSV and explored their underlying mechanisms. An expectation tool to guide doctors in prioritizing tests or assisting parents in deciding on emergency care for their febrile child would be invaluable. Biomarker analysis performed can provide insight on clinical fields.

**Supplementary Information:**

The online version contains supplementary material available at 10.1007/s13534-024-00450-8.

## Introduction

In the northern hemisphere, two respiratory viruses, influenza (IFN) and respiratory syncytial virus (RSV), emerge seasonally during the fall and winter, causing significant morbidity and mortality, particularly among infants and young children [1–4]. In the United States, the Centers for Disease Control and Prevention (CDC) has reported a considerable burden of IFN and RSV. In the 2019–2020 season alone, 16 million individuals sought medical care for IFN related symptoms, while RSV led to hospitalizations of 58,000–80,000 children under 5 years old each year [4–6]. The outbreak of influenza-like illness (ILI) disrupts economies and overwhelms healthcare facilities [7].

It is very important to identify the ILI state early in the disease, as some of them can worsen rapidly, requiring antibacterial or antiviral therapy immediately. However, due to the similarity of symptoms and signs, it is difficult even for doctors to distinguish the state based on clinical manifestations alone [7, 8]. Parents lack the tools to determine whether an urgent hospital visit is necessary for their children, and doctors, especially those who are less experienced or in seasonal challenge, lack guidance on whether to urgently administer antiviral treatments, resulting in variable sensitivity and accuracy [9]. While Rapid Antigen Test (RAT) offer a cheap and rapid screening option for IFN, their low sensitivity limits their recommended use according to CDC guidelines [10, 11]. Polymerase chain reaction (PCR) is considered a reliable diagnostic tool, but it is expensive, time-consuming, and not practical for the rapid identification of epidemic diseases [12, 13].

**Fig. 1:**
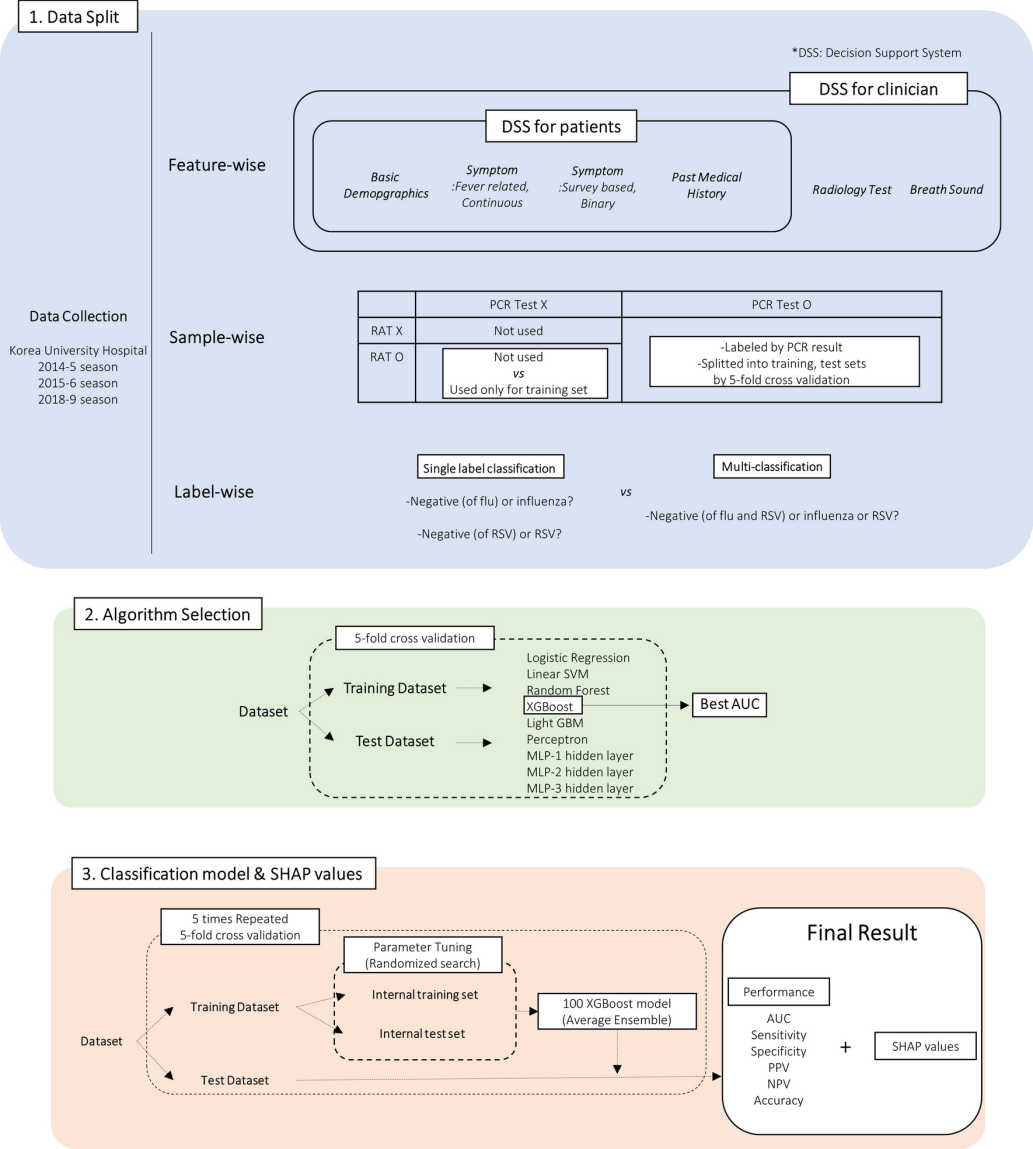
Diagram of research. (1) Data Split, (2) Algorithm Selection, (3) Classification model & SHAP values

In this study, our objective was to develop an AI-assisted diagnostic pipeline that can guide doctors in determining the appropriate test for specific organisms in febrile patients, as well as help parents decide whether their febrile child requires immediate care in the emergency room or hospital. We proceeded to construct two distinct prediction models, as illustrated in Fig. 1. The first model, Decision Support System for Patients (DSS-P), utilized features that could be easily assessed within a household setting, patient demographics, symptoms, and medical history. The second model, Decision Support System for Clinicians (DSS-C), incorporated additional features that are readily available in any hospital, radiology test, and breath sound results. We were able to compare in which experiment setting shows the best performance in each performance index. Our study went beyond merely identifying biomarkers; we utilized Shapley Additive exPlanations (SHAP) values to conduct a deep analysis of the roles and interactions of key features, offering a comprehensive understanding that supports both diagnostic decision-making and the development of targeted treatments.

In Sect. 2. *Literature Survey*, we investigated recent studies related on *A. ILI prediction* and *B. Machine learning and interpretation*. In Sect. 3. *Proposed System*, we introduced the design of studies concretely in methodological manner. We firstly introduced the dataset we collected and utilized for the system in *A. Data Collection and Data Characteristics*. As illustrated in *B. Selection of machine learning algorithm*, we suggested to decide the best performing ML algorithm before building DSS. We compared the nine different machine learning algorithms, and observed that XGBoost algorithms performed better than others significantly. As organized in *C. Data Division and Optimization of XGBoost algorithm*, by XGBoost model with parameter tuning and averaging ensemble, we experimented with different settings in 1) sample-wise (whether to put samples tested only by RAT into training) and 2) label-wise (whether to consider both diseases together). After the prediction, we implemented the analysis on important features as in *D. Interpretation (Shapley Additive exPlanations)*. In Sect. 4. *Results*, we arranged the performance and interpretation results from DSS-P and DSS-C, separately for *A. Influenza* and *B. RSV.* Section 5. *Discussion* summarizes the outline of the paper and deeply explain the pattern appeared in Results. Section 6. *Conclusion* highlights major contribution provided by this paper.

## Literature survey

### Literatures on ILI prediction

With the recent advancements in machine learning, researchers have developed prediction models for ILI. Hung et al. [14] constructed an XGBoost model for predicting IFN, while Tso et al. [15] also utilized an XGBoost model for RSV, achieving Area under ROC Curve (AUC) values of 0.82 and 0.919 respectively. These models incorporated features, such as patient demographics, medical history, symptoms, signs, the week of the IFN season and electronic health records. However, features that require significant cost and time in hospitals did not prove advantageous as auxiliary tools prior to PCR testing. Notably, no studies have simultaneously examined IFN and RSV from the same samples, despite the close relationship between these diseases. Additionally, as machine learning is to be used as an assistive tool, the model should be adaptable to various settings, such as being specialized for parents or clinicians. This adaptability has not yet been reported in the literature.

### Literatures on machine learning and interpretation

In this study, we utilized basic algorithms such as tree ensembles and MLP. Although many advanced deep learning algorithms have been proposed recently, we opted to experiment with widely known and commonly used algorithms. While some deep learning networks may outperform tree ensembles, they often come at the cost of reduced interpretability. Moreover, there is no clear baseline network for tabular datasets [16]. In fact, tree ensemble methods frequently outperform deep learning networks in these scenarios [17]. Given this context, we determined that focusing on popular algorithms would be more beneficial.

In future work, we plan to implement recent deep learning networks specifically designed for tabular datasets. For example, the FT-Transformer employs a ‘feature tokenizer’ to transform features into embeddings, which are then processed by a transformer. This embedding process enables the network to efficiently handle different types of features, both numerical and categorical [16]. Additionally, the Self-Attention and Intersample Attention Transformer (SAINT) has demonstrated high performance by utilizing self-attention and intersample attention, capturing interactions during the decision process [19].

Furthermore, it is important to discuss the validity of the interpretation. While SHAP is a widely recognized interpretation method in medical informatics, the validity of interpretations remains an issue under debate in the machine learning field. To support our interpretation, we conducted experiments using only the top five features from the best-performing set in each DSS and for each disease. As shown in Supplementary Table 9, the performance decreased compared to using all features, as expected. However, the difference was not substantial, indicating that these top five features contributed the most to the prediction. Although features beyond the top five still hold valuable information, the top five features presented in the Results section should be prioritized in data collection strategies. Additionally, we conducted statistical analyses when interpreting SHAP dependence plots [18, 20], filtering results to include only those that were statistically significant. Moreover, while adding RAT samples improved performance in some indices, it did not lead to improvements across all indices, which means that the interpretation may not suffer from the lack of data sizes or performance [21].

## Proposed system

The pipeline of the overall system is illustrated in Fig. 1. After collecting datasets (3.A) and selecting the best-performing algorithm (3.B), 100 XGBoost models were built for each dataset, and an average ensemble was used to evaluate performance (3.C). Finally, SHAP analysis was applied to the best model identified in the previous stage (3.D).

###  Data collection

The research protocol received approval from the Institutional Review Board (IRB) of the Korea University Ansan Hospital, under approval numbers 2014AS0040, 2017AS0712, and 2018AS0253. Data collection occurred over three periods among: 2014–2015, 2015–2016, and 2018–2019, with sample sizes of 1015, 1135, and 409 individuals, respectively. Data was collected from three sectors: outpatients, inpatients and emergency room. All samples underwent testing using PCR, RAT (RAT for IFN only), or both. As detailed in Supplementary Table 1, samples were categorized by data type and variable values based on the presence of IFN and RSV. In the PCR dataset, the statuses of IFN and RSV were determined based solely on PCR results. For samples where RAT was performed but PCR was not, IFN status was determined by the RAT result, while RSV was labeled as negative only when IFN was positive by RAT. Since PCR is the definitive test confirming ILI, the test set exclusively contained PCR data. The performance of RAT was detailed in Supplementary Table 2.

As illustrated in Supplementary Table 1, in DSS-P, 20 features including basic demographics, symptoms, and past medical history were utilized, while the DSS-C employed 24 features, adding information of breath sounds (rale/crackle, wheezing/chest retraction, coarse/stridor) and chest X-rays. Except for age, sex, peak body temperature, and fever duration before the visit, features had missing values in samples, due to “unknown” for symptoms and medical history and untested for breath sound assessments or chest X-rays. Statistical analysis aimed to determine if the differences between the feature values of the negative and positive groups were statistically significant. For continuous features such as age, peak body temperature, and fever duration, a t-test was applied (Levine test with *p*-value of 0.05 was performed to decide whether the variance was equal) if the distribution in both groups was normal (*p* > 0.05 in the Shapiro–Wilk test), while the Mann–Whitney U test was used when normality was not achieved. For nominal features, a chi-square test was employed. Most features showed statistical significance, with exceptions including fever duration before the visit and convulsion in IFN, sex and rale/crackle in RSV.

### Selection of machine learning algorithm

To determine the best machine learning algorithm for the task, we tested several machine learning algorithms as illustrated in 2. Algorithm Selection in Fig. 1. The algorithms evaluated were: (1) Logistic Regression (2) Linear Support Vector Machine (3) Random Forest (4) eXtreme Gradient Boosting (XGBoost) (5) Light Gradient Boosting Machine (LGBM) (6) Perceptron (7) Multi-Layer Perceptron with 1 hidden layer, (8) 2 hidden layers (9) 3 hidden layers. To ensure more generalized performance, every performance was calculated using fivefold cross-validation. To tune the hyperparameters of the models, another round of fivefold cross-validation was performed, using only the training dataset within each fold. By grid search of parameter grid, listed in Supplementary 1. Hyperparameter Details for machine learning model, the parameter set that yielded the best AUC was selected as the optimal configuration.

The AUC was utilized to select the most effective algorithm, and the model was evaluated for its ability to predict IFN and RSV using four distinct setups: (1) DSS-P for IFN, (2) DSS-C for IFN, (3) DSS-P for RSV, and (4) DSS-C for RSV. All analyses were conducted using datasets tested by PCR. Before training, datasets were normalized using the standard scaler. Missing values were imputed using Multivariate Imputation by Chained Equations [22]. Results were evaluated based on the average AUC reported in Supplementary Table 3 and the statistical significance of differences (*p*-value) shown in Supplementary Table 4. XGBoost and LGBM demonstrated the highest AUC across all experiments, with statistical significance (all *p*-values < 0.01, except for one scenario with MLP-1 hidden layer where *p* < 0.1). Given that XGBoost and LGBM are both tree-based ensemble methods and XGBoost showed higher AUC in two experiments, identical in one, lower in one, we chose XGBoost for the decision support system.

### Data division and optimization of XGBoost algorithm

As illustrated in Fig. 1, ‘Data Split’, the experiments utilized different sample allocations: (1) feature-wise, (2) sample-wise, and (3) label-wise. The DSS-P and DSS-C models were separated feature-wise. In the sample-wise approach, samples tested by RAT but not by PCR were either added to or excluded from the training datasets. When predicting only IFN, all samples tested by RAT can be used, as the state of IFN was indicated. However, for RSV, only samples that tested positive for IFN can be added for training, since only IFN positivity can inform RSV negativity. In the label-wise approach, the XGBoost model was built to predict either a single disease (positive vs. negative for IFN and positive vs. negative for RSV) or two diseases simultaneously (positive for IFN vs. positive for RSV vs. negative for both).

In each setting, an XGBoost model was constructed, as detailed in the "3. Classification model & SHAP values" section of Fig. 1. XGBoost allows missing values to be treated as an independent category, facilitating the handling of incomplete data. To ensure robust comparisons, we performed five repetitions of stratified fivefold cross-validation, as multiple performance scores are required to stably compare results. Parameter tuning was conducted within each fold’s training dataset using a randomized search across a predefined grid, detailed in "Supplementary 1. Hyperparameter Details for machine learning model”. The optimal classification threshold to calculate performance indexes, sensitivity, specificity, Positive Predictive Values (PPV) and Negative Predictive Values (NPV), was established using J-statistics on fivefold cross validation of training dataset [23]. Ultimately, 100 XGBoost models with different random states were built, and their predictive outputs were averaged to create a final predictive value through an average ensemble approach.

###  Interpretation (shapley additive explanations)

**Fig. 2:**
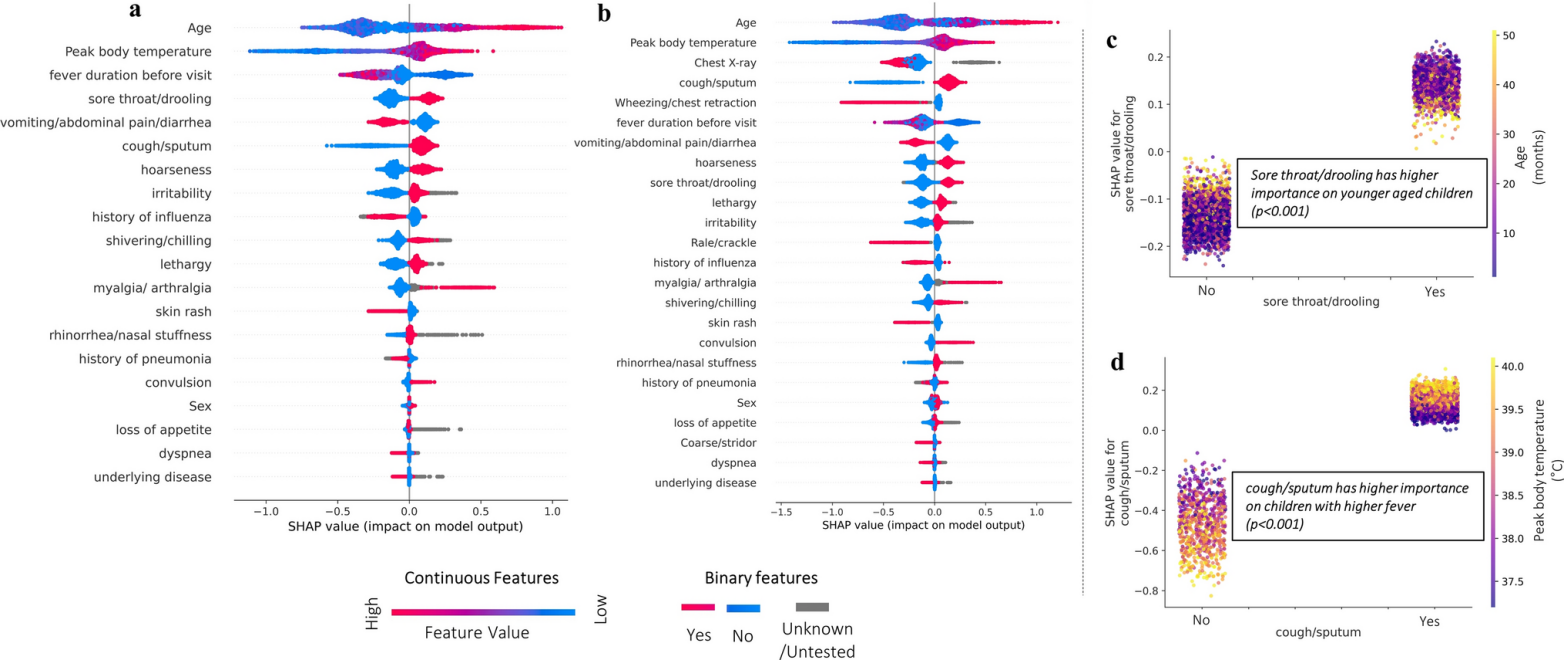
Biomarkers for IFN in each system identified by machine learning. **a** SHAP summary plot in DSS-P, **b** SHAP summary plot in DSS-C, **c** SHAP Dependence plot of sore throat/drooling in DSS-P, **d** SHAP Dependence plot of cough/sputum in DSS-C. SHAP values were obtained from the best performing set in Table 1, which is PCR and RAT samples as training for predicting IFN only in both DSS-P and DSS-C. SHAP values were collected from test set in each fold cross validation

**Fig. 3:**
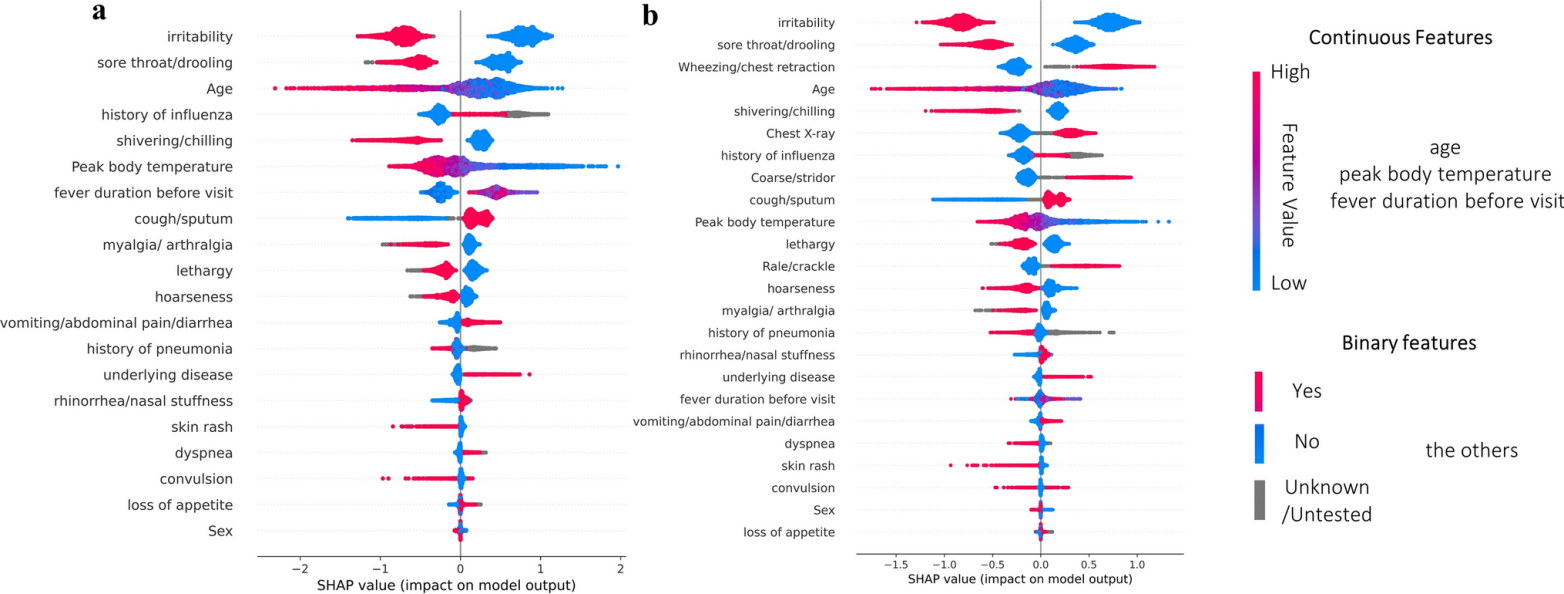
Biomarkers for RSV in each system identified by machine learning. SHAP summary plot in **a** DSS-P and **b** DSS-C. SHAP values were obtained from the best performing set by AUC in Table 3, which is PCR + RAT as training for predicting RSV only in DSS-P and predicting RSV only trained by PCR samples only in DSS-C. SHAP values were collected from test set in each fold cross validation

**Fig. 4:**
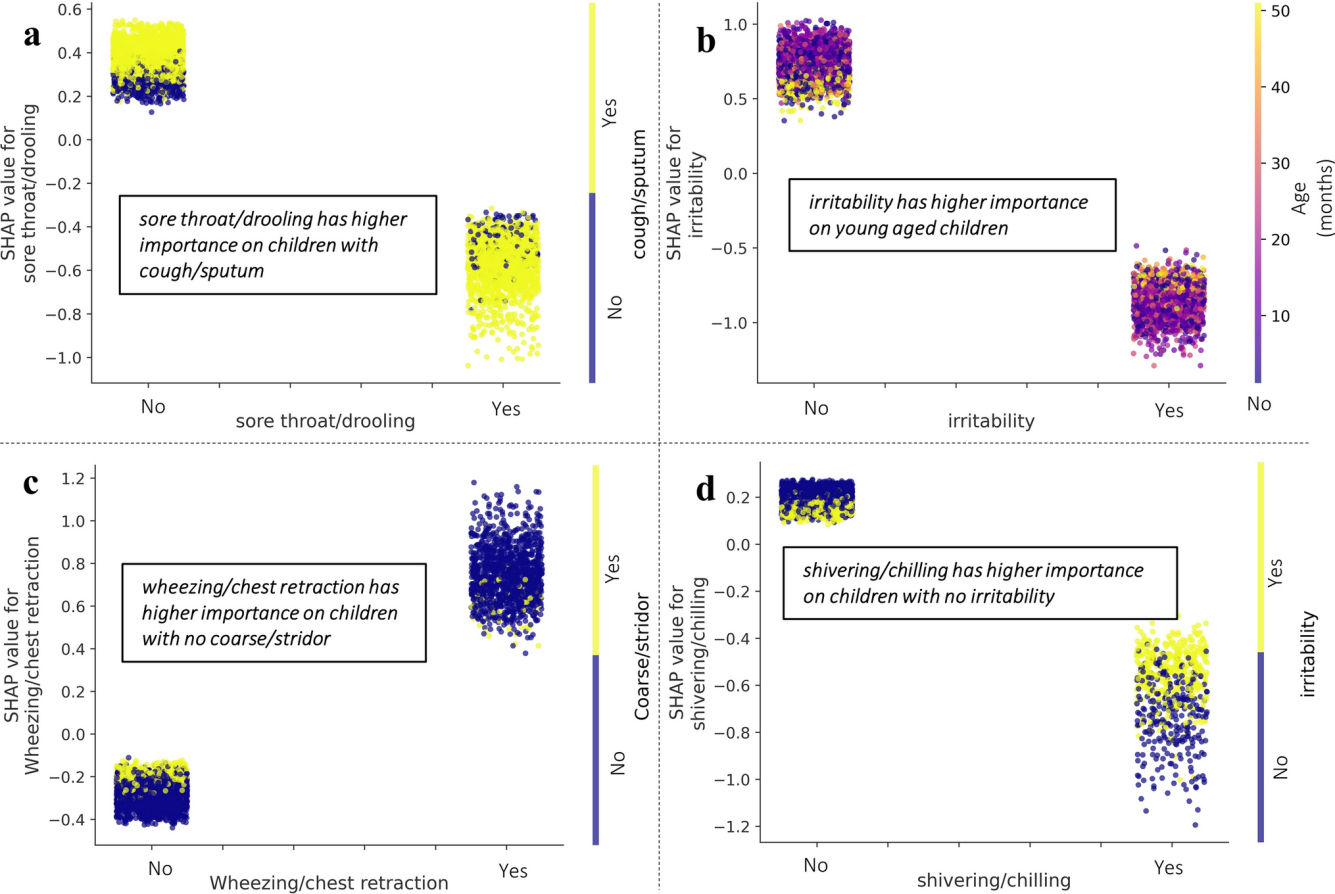
Biomarkers for RSV in each system identified by machine learning. SHAP Dependence plot of **a** sore throat/drooling, **b** irritability, **c** wheezing/chest retraction, **d** shivering/chilling in DSS-C. SHAP values were obtained from the best performing set by AUC in Table 3, which is PCR + RAT as training for predicting RSV only in DSS-P and predicting RSV only trained by PCR samples only in DSS-C. SHAP values were collected from test set in each fold cross validation

SHAP analysis, grounded in game theory, was utilized to unravel how the top-performing machine learning algorithm predicts outcomes. This model-agnostic approach allows for an examination of how individual features in samples were used for predictions. SHAP not only ranks features by their importance but also illustrates their distribution and interactions with other features [24]. SHAP values were generated from every test set in the model of optimal experimental settings (highest average AUC) for each disease (IFN and RSV) in each DSS. SHAP values were collected in every training, not averaged as predictive values in 100 averaging ensembles. These values are visualized in SHAP summary plots, presented in Figs. 2 and 3. In these plots, the sequence of features reflects their rank based on the average of absolute SHAP values. Each dot represents a sample; its color shows the feature value, location indicates the SHAP value, and the size signifies the feature’s importance. The sign of the SHAP value shows the direction of its impact on the prediction: a positive sign suggests an increased likelihood of a positive outcome, and a negative sign indicates the opposite. In Figs. 2 and 4, SHAP dependence plots further clarify the interactions utilized by the machine learning model. The x-axis for its feature value and the y-axis for its SHAP value. The color in the dependence plot signifies the value of the interacting feature, providing insights into how feature interactions influence the prediction.

When the SHAP values are clearly divided by feature value, indicating that the function is interpretable, it demonstrates that the model has recognized the importance of the feature. In Figs. 2 and 3, most features exhibited a clear sign division of SHAP values by their feature values, corresponding to the results, where most of features were statistically significant, shown in Supplementary Table 1. While providing the function of all features recognized by the XGBoost model in SHAP summary plots, we focused on the analysis of the top five features in this paper for practicality. We considered the convenience that parents and clinicians may experience, which would diminish with the consideration of additional biomarkers.

## Results

### Influenza

####  Performance

Table 1 illustrates the results of each experiment conducted on IFN prediction. Supplementary Tables 5 and 6 demonstrate the statistical significance of differences between these experiments, calculated using the Wilcoxon Rank Sum Test. In the DSS-P segment, the model trained with samples tested by PCR or RAT, predicting IFN only, exhibited the best performance in terms of AUC, specificity, and PPV, achieving statistical significance (*p* < 0.05). However, for sensitivity and NPV, the model trained solely with PCR samples to classify both IFN and RSV together showed superior performance. In the DSS-C segment, the best combination improved the average AUC by 0.027 compared to DSS-P. The model trained with samples tested by PCR or RAT and predicting IFN only showed the top scores in AUC (*p* < 0.05 in all cases) and PPV. However, the highest specificity was seen in the model trained with both PCR and RAT samples, classifying IFN and RSV together, while the highest sensitivity and NPV were achieved by the model trained solely with PCR samples and classifying IFN only.

**Table 1:** Performance of the machine learning model for IFN prediction

Type of system	Decision support system for patients				Decision support system for clinicians			
Training data/Test data	PCR/PCR		PCR + RAT/PCR		PCR/PCR		PCR + RAT/PCR	
Performance	IFN	IFN, RSV	IFN	IFN, RSV	IFN	IFN, RSV	IFN	IFN, RSV
AUC	0.728 (0.69–0.76)	0.718 (0.64–0.79)	**0.749 (0.70–0.80)**	0.710 (0.64–0.78)	0.747 (0.70–0.80)	0.736 (0.64–0.83)	**0.776 (0.73–0.82)**	0.740 (0.68–0.80)
Sensitivity	0.775 (0.65–0.90)	**0.800 (0.71–0.89)**	0.625 (0.55–0.70)	0.688 (0.61–0.77)	**0.662 (0.41–0.92)**	**0.662 (0.43–0.90)**	0.537 (0.41–0.67)	0.412 (0.20–0.63)
Specificity	0.585 (0.51–0.66)	0.554 (0.45–0.66)	**0.742 (0.70–0.78)**	0.597 (0.50–0.69)	0.670 (0.57–0.77)	0.646 (0.54–0.76)	0.774 (0.72–0.83)	**0.806 (0.76–0.85)**
PPV	0.149 (0.14–0.16)	0.146 (0.12–0.17)	**0.187 (0.15–0.23)**	0.139 (0.11–0.16)	0.156 (0.14–0.18)	0.150 (0.12–0.18)	**0.183 (0.14–0.23)**	0.160 (0.12–0.20)
NPV	0.967 (0.95–0.98)	**0.968 (0.96–0.98)**	0.955 (0.94–0.97)	0.953 (0.94–0.96)	**0.957 (0.93–0.98)**	0.955 (0.92–0.99)	0.947 (0.93–0.96)	0.937 (0.92–0.96)

The data represents average values with 95% confidence intervals (CIs). Performance scores were derived from the results of the XGBoost model in each fold of a 5-times repeated stratified fivefold cross-validation, yielding 25 scores per metric. In the ‘classification’ row, the model predictions were categorized into two scenarios: (1) IFN versus negative of IFN, and (2) IFN versus RSV versus negative of both IFN and RSV. Performance was calculated by considering only the predictive value for IFN. The best-performing combination for each performance index and Decision Support System is highlighted in bold.

#### Interpretation

Important indicators identified by the machine learning model are illustrated in Fig. 2. Figure 2a reveals the top five features in the DSS-P system: age, peak body temperature, duration of fever before visit, sore throat/drooling, and vomiting/abdominal pain/diarrhea. In Fig. 2b, these features are shown to retain their importance in the DSS-C system, with Chest X-ray and wheezing/chest retraction added as the third and fifth most significant features, respectively. Across both systems, older age, higher peak body temperature, shorter duration of fever before the visit, the presence of sore throat/drooling, and cough/sputum, as well as the absence of vomiting/abdominal pain/diarrhea, are associated with an increased likelihood of IFN. For the additional features in DSS-C, abnormalities in Chest X-ray and wheezing/chest retraction breath sounds are associated with a decreased likelihood of IFN. Figures 2c, d further highlight binary features in rank of top five that exhibit significant interactions (*p*-value < 0.001 via Pearson correlation analysis) with other variables: sore throat/drooling is more significant in younger children (correlation = 0.31/0.38 for No/Yes cases), while cough/sputum is particularly important for children with higher fevers (correlation = 0.57/0.64 for No/Yes cases).

### RSV

#### Performance

Performance for predicting RSV is detailed in Table 2, with statistical significance assessed using the Wilcoxon Rank Sum Test (Supplementary Tables 7 and 8). Differences of AUC by data sample selection and classification targets were not statistically significant as in IFN, as all comparisons yielded *p*-values greater than 0.05. However, performance varied across other indices. In DSS-P, the model trained by PCR + RAT and predicting RSV only showed the best performance in AUC, sensitivity and NPV, while the model trained by PCR data only and predicting IFN and RSV together showed the best in specificity and PPV. In DSS-C, the model trained by PCR + RAT and predicting RSV only consistently showed the best performance in sensitivity and NPV, but other indexes (AUC, specificity and PPV) were highest in the model trained by only PCR dataset and predicting RSV only. Inclusion of clinical features showed the improvement of AUC with 0.017 by comparing the best average AUC in each DSS.

**Table 2:** Performance of the machine learning model for RSV prediction

Type of system	Decision support system for patients				Decision support system for clinicians			
Training data/Test data	PCR/PCR		PCR + RAT/PCR		PCR/PCR		PCR + RAT/PCR	
Performance	Classification							
	RSV	IFN, RSV	RSV	IFN, RSV	RSV	IFN, RSV	RSV	IFN, RSV
AUC	0.904 (0.87–0.94)	0.901 (0.87–0.94)	**0.907 (0.88–0.93)**	0.904 (0.87–0.94)	**0.924 (0.90–0.95)**	0.915 (0.89–0.94)	**0.924 (0.90–0.95)**	0.918 (0.90–0.94)
Sensitivity	0.853 (0.77–0.93)	0.808 (0.73–0.89)	**0.861 (0.79–0.93)**	0.850 (0.77–0.93)	0.832 (0.74–0.93)	0.834 (0.75–0.92)	**0.884 (0.83–0.94)**	0.858 (0.82–0.90)
Specificity	0.823 (0.77–0.87)	**0.848 (0.80–0.89)**	0.817 (0.77–0.86)	0.805 (0.76–0.85)	**0.875 (0.80–0.95)**	0.844 (0.81–0.87)	0.787 (0.72–0.85)	0.826 (0.78–0.87)
PPV	0.768 (0.72–0.81)	**0.784 (0.72–0.84)**	0.764 (0.73–0.80)	0.748 (0.71–0.79)	**0.825 (0.76–0.89)**	0.786 (0.77–0.80)	0.741 (0.68–0.80)	0.772 (0.73–0.82)
NPV	0.893 (0.84–0.94)	0.867 (0.82–0.92)	**0.898 (0.85–0.94)**	0.889 (0.84–0.94)	0.887 (0.83–0.94)	0.884 (0.84–0.93)	**0.910 (0.87–0.95)**	0.896 (0.87–0.92)

The data represent average values with 95% CIs. Performance scores were derived from the results of the XGBoost model in each fold of a 5-times repeated stratified fivefold cross-validation, yielding 25 scores per metric. In the ‘classification’ row, the model predictions were categorized into two scenarios: (1) RSV versus negative of RSV, and (2) IFN versus RSV versus negative of IFN and RSV. Performance was calculated by considering only the predictive value for RSV. The best-performing combination for each performance index and Decision Support System is highlighted in bold.

#### Interpretation

In Figs. 3 and 4, features were ranked by their importance in the machine learning model. In DSS-P (Fig. 3a), the top five features were irritability, sore throat/drooling, age, history of influenza and shivering/chilling. In DSS-C (Fig. 3b), the top features were irritability, sore throat/drooling, wheezing/chest retraction, age, and shivering/chilling. The model identified parameters that increase the likelihood of RSV to include the absence of irritability, sore throat/drooling and shivering/chilling, younger age, a history of IFN, and wheezing/chest retraction sounds. Binary features among the top five were selected based on their clear interaction with other features. As the interaction of these features showed the same pattern between DSS-P and DSS-C, feature overlapped were analyzed by the SHAP dependence plot in DSS-C. These interactions were significant (*p* < 0.001) according to the t-test/ Wilcoxon Rank Sum Test for Fig. 4a, c and d, and Pearson correlation analysis for Fig. 4b. In Fig. 4a, sore throat/drooling was deemed less important in cases with no cough/sputum. Figure 4b shows that irritability is a more critical indicator in younger children compared to older children, with correlations of -0.32 and -0.23 for No/Yes cases, respectively. In Fig. 4c, wheezing/chest retraction was considered a less essential clue when coarse/stridor breath sounds were observed. Lastly, shivering/chilling was found to be more significant when irritability was not expressed.

## Discussion

In this research, we established the applicable level of performance for both IFN and RSV using readily obtainable features from home settings (20 features, including basic demographics, symptoms, and past medical history) and hospital settings (24 features, adding breath sounds and chest X-ray). In the house setting, the DSS-P achieved AUCs of 0.749 and 0.907 for IFN and RSV, respectively. In the hospital setting, the DSS-C provided AUCs of 0.776 and 0.924 for IFN and RSV, respectively. It is worth noting that not all features need to be present, as this dataset had missing values except for age, sex, and fever duration before visit. The XGBoost model was robust enough to handle missing features and make decisions based on available data. Furthermore, the model outputs a predictive value between zero to one, indicating the likelihood of the prediction’s accuracy. Considering that the model should be used as an assistive tool for patients and clinicians, this predictive value can guide the level of credibility that patients and doctors place on each prediction.

Comparing DSS-P and DSS-C, both IFN and RSV clearly benefited from the inclusion of chest X-ray and breath sound information. In each experimental setting—whether incorporating samples tested only by RAT for training, or predicting two ILI diseases together or individually—different strengths emerged across various performance indices. Generally, adding RAT-based samples to the training dataset increased performance (AUC), especially for IFN (*p* < 0.05). Given that the number of RAT-based samples was limited to IFN-positive when considering RSV, this difference was anticipated. In terms of performance indices, sensitivity and NPV for IFN, and specificity and PPV for RSV, did not improve with RAT-based samples; they slightly decreased instead. RAT based samples appeared to decrease the false positives (FP) for IFN and false negatives (FN) for RSV, but increase the FN for IFN and FP for RSV. The benefits of classifying two ILI diseases together also varied depending on the setting and target, indicating that while it can reduce FP or FN, it might sometimes increase them due to the inclusion of an unnecessary target. The overall results suggest that collecting more data in a balanced composition will likely improve performance, and this research provides an effective list of features for predicting ILI. Until performance stabilizes with a sufficient number of samples, utilizing multiple experimental settings and outputs may provide the necessary insights.

To the best of the authors’ knowledge, this research represents the first study to construct a machine learning model for both IFN and RSV using the same dataset, enabling a comparative analysis of indicators. The need to analyze these two ILI diseases together was underscored by our findings, as most of features displayed opposing patterns between IFN and RSV. For example, age emerged as a significant factor in the prognosis of both diseases—ranking first in the case of IFN and third and fourth in the respective DSS models for RSV. Notably, older children were found to be more susceptible to IFN, while younger children were more vulnerable to RSV. Considering all features where the SHAP values were clearly divided by feature value, only cough/sputum, skin rash, rhinorrhea/nasal stuffiness, and dyspnea showed the same direction of impact. Except for cough/sputum, which increases the likelihood of both IFN and RSV, other features were ranked very low, below average in every DSS model. The opposite pattern of biomarkers indicates that the machine learning model utilized some indicators as the clue of negativity as it works for the clue of other diseases. Given the potential for other diseases to occur within the same period of IFN and RSV, the ranking of features can highlight the unique characteristics of each disease, emphasizing the importance of focusing on top ranked biomarkers.

In the SHAP summary plots of Figs. 2 and 3, even the same feature values displayed a wide range of SHAP values, mostly in the same direction but varying in magnitude. This variation indicates that the machine learning model recognized interactions between features, as partially illustrated in the SHAP dependence plots in Figs. 3. Although not all interactions were interpretable (*p* > 0.05), many of the high-ranked features exhibited intuitive patterns of interaction. These interactions explain why the machine learning model provides a better fit for this dataset than traditional regression analysis would. Considering that less-experienced doctors, or those facing seasonal challenges, might be prone to overlooking complex symptoms in favor of simpler ones, it is crucial to demonstrate how interactions work in an intuitive manner. This can enhance immediate but more accurate responses in medical sites before actually utilizing the real-time prediction system by machine learning.

## Conclusion

Among current machine learning studies on IFN and RSV, this study offers several unique perspectives. We applied machine learning to these two highly related diseases together and built a decision support system in two stages (for parents and for doctors). We also investigated whether including RAT samples would improve predictions. While achieving optimal performance in each stage, we analyzed biomarkers for each experiment, which can be further studied in pediatrics.

## Supplementary Information

Below is the link to the electronic supplementary material.

Supplementary file1 (DOCX 58 KB)

## References

[1] Nairz M, Todorovic T, Gehrer CM, et al. ‘Single-center experience in detecting influenza virus, rsv and sars-cov-2 at the emergency department.’ Viruses. 2023;15(2):470. 10.3390/v15020470. 36851685 10.3390/v15020470 PMC9958692

[2] Griffin MR, Coffey CS, Neuzil KM, Mitchel EF, Wright PF, Edwards KM. ‘Winter viruses: influenza-and respiratory syncytial virus–related morbidity in chronic lung disease.’ Arch Intern Med. 2002;162(11):1229–36. 10.1001/archinte.162.11.1229. 12038940 10.1001/archinte.162.11.1229

[3] Kayode AJ, Banji-Onisile FO, Olaniran AO, Okoh AI. ‘An overview of the pathogenesis, transmission, diagnosis, and management of endemic human coronaviruses: a reflection on the past and present episodes and possible future outbreaks.’ Pathogens. 2021;10(9):1108. 34578140 10.3390/pathogens10091108 PMC8470645

[4] Yaron-Yakoby H, Sefty H, Pando R, et al. ‘Effectiveness of influenza vaccine in preventing medically attended influenza virus infection in primary care, israel, influenza seasons 2014/15 and 2015/16.’ Euro Surveill. 2018;23(7):17–26. 10.2807/1560-7917.ES.2018.23.7.17-00026. 10.2807/1560-7917.ES.2018.23.7.17-00026 PMC5824129 29471622

[5] Paget J, Spreeuwenberg P, Charu V, et al. ‘Global mortality associated with seasonal influenza epidemics: new burden estimates and predictors from the glamor project.’ J Glob Health. 2019. 10.7189/jogh.09.020421. 10.7189/jogh.09.020421 PMC6815659 31673337

[6] Centers for Disease Control and Prevention (2022). Disease burden of flu. Retrieved 0627, 2023, from https://www.cdc.gov/flu/about/burden/index.html

[7] Rha B, Curns AT, Lively JY, et al. ‘Respiratory syncytial virus–associated hospitalizations among young children: 2015–2016.’ Pediatrics. 2020. 10.1542/peds.2019-3611. 10.1542/peds.2019-3611 PMC12874392 32546583

[8] Chowell G, Nishiura H, Bettencourt LM. ‘Comparative estimation of the reproduction number for pandemic influenza from daily case notification data.’ J R Soc Interface. 2007;4(12):155–66. 10.1098/rsif.2006.0161. 17254982 10.1098/rsif.2006.0161 PMC2358966

[9] Campe H, Heinzinger S, Hartberger C, Sing A. ‘Clinical symptoms cannot predict influenza infection during the 2013 influenza season in bavaria, germany.’ Epidemiol Infect. 2016;144(5):1045–51. 10.1017/S0950268815002228. 26388141 10.1017/S0950268815002228

[10] Lam P-P, Coleman BL, Green K, et al. ‘Predictors of influenza among older adults in the emergency department.’ BMC Infect Dis. 2016;16:1–9. 10.1186/s12879-016-1966-4. 27793117 10.1186/s12879-016-1966-4 PMC5084347

[11] Ebell MH, Afonso A. ‘A systematic review of clinical decision rules for the diagnosis of influenza.’ Ann Fam Med. 2011;9(1):69–77. 10.1370/afm.1192. 21242564 10.1370/afm.1192 PMC3022049

[12] Centers for Disease Control and Prevention. Guide for considering influenza testing when influenza viruses are circulating in the community, https://www.cdc.gov/flu/professionals/diagnosis/consider-influenza-testing.htm;/2021 [accessed 10 May 2021].

[13] Dugas AF, Valsamakis A, Atreya MR, et al. ‘Clinical diagnosis of influenza in the ed.’ Am J Emerg Med. 2015;33(6):770–5. 10.1016/j.ajem.2015.03.008. 25827595 10.1016/j.ajem.2015.03.008 PMC4458223

[14] Hung S-K, Wu C-C, Singh A, et al. ‘Developing and validating clinical features-based machine learning algorithms to predict influenza infection in influenzalike illness patients.’ Biomed J. 2023;46(5):100–561. 10.1016/j.bj.2022.09.002. 36150651 10.1016/j.bj.2022.09.002 PMC10498408

[15] Tso CF, Lam C, Calvert J, Mao Q. ‘Machine learning early prediction of respiratory syncytial virus in pediatric hospitalized patients.’ Front Pediatr. 2022;10:886–212. 10.3389/fped.2022.886212. 10.3389/fped.2022.886212 PMC9385995 35989982

[16] Gorishniy Y, et al. Revisiting deep learning models for tabular data. Advan Neural Inf Process Syst. 2021;34:18932–43.

[17] Grinsztajn L, Oyallon E, Varoquaux G. Why do tree-based models still outperform deep learning on typical tabular data? Adv Neural Inf Process Syst. 2022;35:507–20.

[18] Lee Y, Kim K, Seo J. CLE-SH: Comprehensive Literal Explanation package for SHapley values by statistical validity. arXiv preprint arXiv. 2024:2409.12578

[19] Somepalli G, Goldblum M, Schwarzschild A, Bruss CB, Goldstein T. Saint: Improved neural networks for tabular data via row attention and contrastive pre-training. arXiv preprint arXiv. 2021:2106.01342

[20] Lee Y, Seo J. Suggestion of statistical validation on feature importance of machine learning. In: 2023 45th Annual International Conference of the IEEE Engineering in Medicine & Biology Society (EMBC) 2023 Jul 24 (pp. 1-4). IEEE. 10.1109/EMBC40787.2023.10340208 38083557

[21] Lee Y, Baruzzo G, Kim J, Seo J, Di Camillo B. Validity of Feature Importance in Low-Performing Machine Learning for Tabular Biomedical Data. arXiv preprint arXiv. 2024:2409.13342

[22] Azur MJ, Stuart EA, Frangakis C, Leaf PJ. ‘Multiple imputation by chained equations: What is it and how does it work?’ Int J Methods Psychiatr Res. 2011;20(1):40–9. 10.1002/mpr.329. 21499542 10.1002/mpr.329 PMC3074241

[23] Fernández A, García S, Galar M, et al. ‘Data intrinsic characteristics.’ Learn Imbalanced Data Sets. 2018. 10.1007/978-3-319-98074-4_10.

[24] Lundberg SM, Lee S-I. ‘A unified approach to interpreting model predictions.’ Advan Neural Inf process Syst. 2017. 10.48550/arXiv.1705.07874.

[25] OpenAI, Chatgpt: An ai language model based on the gpt-4 architecture, Accessed: 2024–06–21, 2023. [Online]. Available: https://www.openai.com/chatgpt.

