# Supplementary material for: AI-assisted diagnostic approach for the influenza-like illness in children: decision support system for patients and clinicians
Source: Biomed Eng Lett. 2024 Dec 30;15(2):327–36. doi: 10.1007/s13534-024-00450-8 (PMC11871169; doi:10.1007/s13534-024-00450-8)
Supplement: Supplementary file 1 — Supplementary file1 (DOCX 58 KB) [file 13534_2024_450_MOESM1_ESM.docx]

**Supplementary Material**

AI-assisted diagnostic approach for the influenza-like illness in children

: Decision support system for patients and clinicians

***Tables and Figures are arranged according to the mention in the Main Article.**

|  |  | **IFN** | | | **RSV** | | |
| --- | --- | --- | --- | --- | --- | --- | --- |
|  | **Total** | **Negative** | **Positive** |  | **Negative** | **Positive** |  |
| **Data Type** | 2559 |  |  |  |  |  |  |
| PCR data | 938 | 858 | 80 |  | 558 | 380 |  |
| RAT data without PCR | 1621 | 1219 | 402 |  | 402 | 0 |  |
| **Variable Name** |  |  |  | p-value |  |  | p-value |
| Sex |  |  |  | <0.05 |  |  | 0.09 |
| Female | 432 | 392 (41.8) | 40 (4.3) |  | 265 (28.3) | 167 (17.8) |  |
| Male | 506 | 466 (49.7) | 40 (4.3) |  | 293 (31.2) | 213 (22.7) |  |
| Age | 18.9±20.8 | 18.3±20.3 | 25.8±24.5 | <0.01 | 22.1±23.9 | 14.2±13.9 | <0.001 |
| Peak body temperature | 38.8±0.9 | 38.7±0.9 | 39.0±0.7 | <0.01 | 39.0±0.8 | 38.5±0.9 | <0.001 |
| fever duration before visit | 1.9±2.3 | 1.9±2.3 | 2.0±2.4 | 0.57 | 2.1±2.3 | 1.7±2.3 | <0.01 |
| shivering/chilling |  |  |  | <0.001 |  |  | <0.001 |
| No | 671 | 624 (66.5) | 47 (5.0) |  | 316 (33.7) | 355 (37.8) |  |
| Yes | 195 | 171 (18.2) | 24 (2.6) |  | 175 (18.7) | 20 (2.1) |  |
| lethargy |  |  |  | <0.001 |  |  | <0.001 |
| No | 504 | 478 (51.0) | 26 (2.8) |  | 203 (21.6) | 301 (32.1) |  |
| Yes | 379 | 334 (35.6) | 45 (4.8) |  | 305 (32.5) | 74 (7.9) |  |
| sore throat/drooling |  |  |  | <0.001 |  |  | <0.001 |
| No | 541 | 511 (54.5) | 30 (3.2) |  | 207 (22.1) | 334 (35.6) |  |
| Yes | 299 | 256 (27.3) | 43 (4.6) |  | 263 (28.0) | 36 (3.8) |  |
| irritability |  |  |  | <0.001 |  |  | <0.001 |
| No | 487 | 461 (49.1) | 26 (2.8) |  | 158 (16.8) | 329 (35.1) |  |
| Yes | 405 | 359 (38.3) | 46 (4.9) |  | 359 (38.3) | 46 (4.9) |  |
| rhinorrhea/nasal stuffiness |  |  |  | <0.001 |  |  | <0.001 |
| No | 185 | 175 (18.7) | 10 (1.1) |  | 122 (13.0) | 63 (6.7) |  |
| Yes | 740 | 672 (71.6) | 68 (7.2) |  | 423 (45.1) | 317 (33.8) |  |
| loss of appetite |  |  |  | <0.001 |  |  | <0.001 |
| No | 428 | 404 (43.1) | 24 (2.6) |  | 191 (20.4) | 237 (25.3) |  |
| Yes | 488 | 436 (46.5) | 52 (5.5) |  | 347 (37.0) | 141 (15.0) |  |
| cough/sputum |  |  |  | <0.001 |  |  | <0.001 |
| No | 164 | 155 (16.5) | 9 (1.0) |  | 130 (13.9) | 34 (3.6) |  |
| Yes | 762 | 691 (73.7) | 71 (7.6) |  | 418 (44.6) | 344 (36.7) |  |
| vomiting/abdominal pain  /diarrhea |  |  |  | <0.01 |  |  | <0.001 |
| No | 590 | 543 (57.9) | 47 (5.0) |  | 323 (34.4) | 267 (28.5) |  |
| Yes | 330 | 298 (31.8) | 32 (3.4) |  | 218 (23.2) | 112 (11.9) |  |
| dyspnea |  |  |  | <0.001 |  |  | <0.001 |
| No | 715 | 656 (69.9) | 59 (6.3) |  | 381 (40.6) | 334 (35.6) |  |
| Yes | 141 | 124 (13.2) | 17 (1.8) |  | 104 (11.1) | 37 (3.9) |  |
| myalgia/ arthralgia |  |  |  | <0.001 |  |  | <0.001 |
| No | 675 | 626 (66.7) | 49 (5.2) |  | 315 (33.6) | 360 (38.4) |  |
| Yes | 37 | 32 (3.4) | 5 (0.5) |  | 35 (3.7) | 2 (0.2) |  |
| hoarseness |  |  |  | <0.001 |  |  | <0.001 |
| No | 532 | 502 (53.5) | 30 (3.2) |  | 232 (24.7) | 300 (32.0) |  |
| Yes | 359 | 314 (33.5) | 45 (4.8) |  | 280 (29.9) | 79 (8.4) |  |
| convulsion |  |  |  | 0.31 |  |  | <0.001 |
| No | 830 | 760 (81.0) | 70 (7.5) |  | 467 (49.8) | 363 (38.7) |  |
| Yes | 67 | 62 (6.6) | 5 (0.5) |  | 53 (5.7) | 14 (1.5) |  |
| skin rash |  |  |  | 0.05 |  |  | <0.001 |
| No | 836 | 765 (81.6) | 71 (7.6) |  | 465 (49.6) | 371 (39.6) |  |
| Yes | 86 | 77 (8.2) | 9 (1.0) |  | 77 (8.2) | 9 (1.0) |  |
| history of influenza |  |  |  | <0.05 |  |  | 0.58 |
| No | 614 | 548 (58.4) | 66 (7.0) |  | 435 (46.4) | 179 (19.1) |  |
| Yes | 62 | 57 (6.1) | 5 (0.5) |  | 45 (4.8) | 17 (1.8) |  |
| history of pneumonia |  |  |  | <0.001 |  |  | <0.001 |
| No | 491 | 447 (47.7) | 44 (4.7) |  | 324 (34.5) | 167 (17.8) |  |
| Yes | 209 | 181 (19.3) | 28 (3.0) |  | 167 (17.8) | 42 (4.5) |  |
| underlying disease |  |  |  | <0.001 |  |  | <0.05 |
| No | 845 | 770 (82.1) | 75 (8.0) |  | 513 (54.7) | 332 (35.4) |  |
| Yes | 76 | 72 (7.7) | 4 (0.4) |  | 40 (4.3) | 36 (3.8) |  |
| Chest X-ray |  |  |  | <0.001 |  |  | <0.001 |
| No | 546 | 491 (52.3) | 55 (5.9) |  | 375 (40.0) | 171 (18.2) |  |
| Yes | 340 | 319 (34.0) | 21 (2.2) |  | 154 (16.4) | 186 (19.8) |  |
| Rale/crackle |  |  |  | <0.001 |  |  | 0.65 |
| No | 755 | 686 (73.1) | 69 (7.4) |  | 454 (48.4) | 301 (32.1) |  |
| Yes | 168 | 158 (16.8) | 10 (1.1) |  | 99 (10.6) | 69 (7.4) |  |
| Wheezing/chest retraction |  |  |  | <0.001 |  |  | <0.001 |
| No | 691 | 619 (66.0) | 72 (7.7) |  | 481 (51.3) | 210 (22.4) |  |
| Yes | 232 | 225 (24.0) | 7 (0.7) |  | 72 (7.7) | 160 (17.1) |  |
| Coarse/stridor |  |  |  | <0.001 |  |  | <0.001 |
| No | 763 | 703 (74.9) | 60 (6.4) |  | 476 (50.7) | 287 (30.6) |  |
| Yes | 160 | 141 (15.0) | 19 (2.0) |  | 77 (8.2) | 83 (8.8) |  |

Supplementary Table 1. Characteristics of the subjects according to the state of indicators and ILI.

**Supplementary Section 1. Hyperparameter Details for machine learning algorithm comparison**

All machine learning algorithms were built using the scikit-learn library, with default parameter values prioritized for the choice of parameters.

***Logistic Regression.*** Regularized logistic regression with L2 penalty was used. The optimization was performed using the lbfgs solver with a tolerance parameter of 1e-4. Grid search was conducted to tune the regularization parameter in the grid of $2^{(-5,-3,-1,1,3,5)}$.

***Linear SVM***. To prevent overfitting, the squared L2 penalty was employed. The 'gamma' parameter was set as 1 divided by the product of the number of features and the variation of features (option 'scale' in the function). A grid search was conducted to tune the regularization parameter in the grid of $2^{(-5,-3,-1,1,3,5,7,9,11,13)}$

***Random Forest.*** The split criterion used was the Gini impurity. The maximum depth was set to unlimited to expand nodes until all leaves are pure or have fewer samples than the 'min_samples_split' parameter. 'min_samples_split' and 'min_samples_leaf' were both set to two. The best split was determined by considering the square root of the number of features. The number of estimators was set to 100.

Multi-Layer Perceptron (***MLP***). For the MLP regressor model, the LBFGS optimizer was used to minimize the squared error. The optimization was stopped when the error reached 1e-4. The ReLU activation function was employed. Grid search was performed to optimize the learning rate and L2 regularization parameter in the grids [0.005, 0.01, 0.05, 0.1, 0.5] and [1e-3, 1e-2, 0.1, 1, 10], respectively. The hidden layer size was set to the number of features in each model.

Extreme gradient boosting (***XGBoost***). XGBoost was tuned in two stages. In the first stage, XGBoost was used for selecting the best algorithm (Supplementary Figure 2), and only the learning rate and regularization factor (alpha) were tuned using the same grid as MLP. After selecting XGBoost for building the prediction model, the parameters were tuned using randomized search in each fold of cross-validation for every experiment. Iteration number was 1000. Parameters were tuned from following grids: 1) learning rate (eta): [0.005, 0.01, 0.05, 0.1,0.25,0.5], 2) reg_lambda: [1e-5, 1e-2, 0.1, 1, 100], 3) alpha (L1 regularization term): [1e-3, 1e-2, 0.1, 1, 10], 4) number of estimators: [100,200,300,500], 5) max_depth: [3, 4, 5, 6, 7, 8], 6) gamma: [0,0.1,0.2,0.3,0.4,0.5], 7) subsample: [0.6, 0.7, 0.8, 0.9, 1.0], 8) colsample_bytree: [0.6, 0.7, 0.8, 0.9, 1.0]. While the log-loss function was used to train the XGBoost model, multiclass log loss was employed when considering the two diseases together.

| Performance | | # of samples | |
| --- | --- | --- | --- |
| Sensitivity | 0.835 | TP | 66 |
| Specificity | 0.968 | FP | 24 |
| PPV | 0.733 | TN | 730 |
| NPV | 0.983 | FN | 13 |

Supplementary Table 2. Performance and the number of samples of RAT for the prediction of flu. To identify whether the RAT’s prediction was right or wrong, performance and number of samples were calculated only from samples which had both RAT and PCR result, considering PCR as a ground truth.

|  |  | **Logistic Regression** | **Linear SVM** | **Random Forest** | **XGBoost** | **LightGBM** | **MLP-Perceptron** | **MLP-**  **1 hidden layer** | **MLP-**  **2 hidden layer** | **MLP-**  **3 hidden layer** |
| --- | --- | --- | --- | --- | --- | --- | --- | --- | --- | --- |
| **DSS-P** | **Flu** | 0.647  (0.59-0.70) | 0.501  (0.48-0.52) | 0.499  (0.50-0.50) | 0.685  (0.64-0.73) | 0.700  (0.63-0.77) | 0.563  (0.50-0.62) | 0.682  (0.63-0.73) | 0.595  (0.50-0.69) | 0.651  (0.53-0.77) |
|  | **RSV** | 0.891  (0.88-0.90) | 0.804  (0.78-0.82) | 0.848  (0.82-0.87) | 0.905  (0.90-0.91) | 0.905  (0.89-0.92) | 0.763  (0.71-0.82) | 0.891  (0.88-0.90) | 0.895  (0.89-0.90) | 0.894  (0.89-0.90) |
| **DSS-C** | **Flu** | 0.680  (0.64-0.72) | 0.483  (0.45-0.51) | 0.500  (0.5-0.5) | 0.712  (0.65-0.78) | 0.703  (0.65-0.75) | 0.506  (0.42-0.59) | 0.682  (0.62-0.75) | 0.598  (0.51-0.69) | 0.591  (0.43-0.75) |
|  | **RSV** | 0.912  (0.90-0.92) | 0.838  (0.82-0.86) | 0.873  (0.86-0.88) | 0.926  (0.91-0.94) | 0.921  (0.91-0.93) | 0.773  (0.72-0.82) | 0.913  (0.91-0.92) | 0.914  (0.91-0.92) | 0.916  (0.91-0.93) |

Supplementary Table 3. AUC in each machine learning algorithm. The data represent average values with 95% confidence intervals (CIs). Performance scores were derived from the results of each fold of 5-fold cross validation. All algorithms predicted only a target disease, not the other together.

|  | **Logistic Regression** | **Linear SVM** | **Random Forest** | **XGBoost** | **LightGBM** | **MLP-Perceptron** | **MLP-1 hidden layer** | **MLP-2 hidden layer** | **MLP-3 hidden layer** |
| --- | --- | --- | --- | --- | --- | --- | --- | --- | --- |
| **Logistic Regression** |  | <0.001 | <0.001 | **0.007** | **0.005** | <0.001 | 0.097 | 0.216 | 0.674 |
| **Linear SVM** |  |  | 0.001 | **<0.001** | **<0.001** | 0.658 | <0.001 | <0.001 | <0.001 |
| **Random Forest** |  |  |  | **<0.001** | **<0.001** | 0.177 | <0.001 | <0.001 | <0.001 |
| **XGBoost** |  |  |  |  | **0.927** | **<0.001** | **0.083** | **<0.001** | **0.001** |
| **LightGBM** |  |  |  |  |  | **<0.001** | **0.09** | **<0.001** | **0.017** |
| **MLP-Perceptron** |  |  |  |  |  |  | <0.001 | <0.001 | <0.001 |
| **MLP-1 hidden layer** |  |  |  |  |  |  |  | 0.245 | 0.475 |
| **MLP-2 hidden layer** |  |  |  |  |  |  |  |  | 0.294 |

Supplementary Table 4. Statistical significance (p-value) between performance of machine learning algorithms. The data were collected from AUC values in Supplementary Table 1, 4 experiments x 5 fold cross validation = 20 AUC values in each. Wilcoxon rank sum test was performed to calculate the statistical significance (p-value) of difference between each paired values.

| DSS-P for flu | | PCR | | PCR+RAT as training | |
| --- | --- | --- | --- | --- | --- |
| **AUC** | | Flu | Flu, RSV | Flu | Flu, RSV |
| PCR | Flu |  | p>0.1 | **0.003** | p>0.1 |
|  | Flu, RSV |  |  | **0.022** | p>0.1 |
| PCR+RAT as training | Flu |  |  |  | **0.003** |
|  | Flu, RSV |  |  |  |  |
| DSS-P for flu | | PCR | | PCR+RAT as training | |
| **Sensitivity** | | Flu | Flu, RSV | Flu | Flu, RSV |
| PCR | Flu |  | p>0.1 | **0.001** | p>0.1 |
|  | Flu, RSV |  |  | **0.001** | p>0.1 |
| PCR+RAT as training | Flu |  |  |  | **0.015** |
|  | Flu, RSV |  |  |  |  |
| DSS-P for flu | | PCR | | PCR+RAT as training | |
| **Specificity** | | Flu | Flu, RSV | Flu | Flu, RSV |
| PCR | Flu |  | p>0.1 | **<0.001** | p>0.1 |
|  | Flu, RSV |  |  | **<0.001** | p>0.1 |
| PCR+RAT as training | Flu |  |  |  | **<0.001** |
|  | Flu, RSV |  |  |  |  |
| DSS-P for flu | | PCR | | PCR+RAT as training | |
| **PPV** | | Flu | Flu, RSV | Flu | Flu, RSV |
| PCR | Flu |  | p>0.1 | **<0.001** | 0.067 |
|  | Flu, RSV |  |  | **<0.001** | p>0.1 |
| PCR+RAT as training | Flu |  |  |  | **<0.001** |
|  | Flu, RSV |  |  |  |  |
| DSS-P for flu | | PCR | | PCR+RAT as training | |
| **NPV** | | Flu | Flu, RSV | Flu | Flu, RSV |
| PCR | Flu |  | p>0.1 | p>0.1 | p>0.1 |
|  | Flu, RSV |  |  | 0.081 | p>0.1 |
| PCR+RAT as training | Flu |  |  |  | 0.092 |
|  | Flu, RSV |  |  |  |  |

Supplementary Table 5. Statistical significance of differences in performance scores (DSS-P) for influenza. The Wilcoxon test was used to assess the significance of median differences between groups.

| DSS-C for flu | | PCR | | PCR+RAT as training | |
| --- | --- | --- | --- | --- | --- |
| **AUC** | | Flu | Flu, RSV | Flu | Flu, RSV |
| PCR | Flu |  | p>0.1 | **<0.001** | p>0.1 |
|  | Flu, RSV |  |  | **0.011** | p>0.1 |
| PCR+RAT as training | Flu |  |  |  | **0.014** |
|  | Flu, RSV |  |  |  |  |
| DSS-C for flu | | PCR | | PCR+RAT as training | |
| **Sensitivity** | | Flu | Flu, RSV | Flu | Flu, RSV |
| PCR | Flu |  | p>0.1 | **0.005** | **0.001** |
|  | Flu, RSV |  |  | **0.001** | **<0.001** |
| PCR+RAT as training | Flu |  |  |  | **0.031** |
|  | Flu, RSV |  |  |  |  |
| DSS-C for flu | | PCR | | PCR+RAT as training | |
| **Specificity** | | Flu | Flu, RSV | Flu | Flu, RSV |
| PCR | Flu |  | p>0.1 | **<0.001** | **<0.001** |
|  | Flu, RSV |  |  | **<0.001** | **<0.001** |
| PCR+RAT as training | Flu |  |  |  | **0.016** |
|  | Flu, RSV |  |  |  |  |
| DSS-C for flu | | PCR | | PCR+RAT as training | |
| **PPV** | | Flu | Flu, RSV | Flu | Flu, RSV |
| PCR | Flu |  | p>0.1 | **<0.001** | **0.01** |
|  | Flu, RSV |  |  | **<0.001** | **0.006** |
| PCR+RAT as training | Flu |  |  |  | p>0.1 |
|  | Flu, RSV |  |  |  |  |
| DSS-C for flu | | PCR | | PCR+RAT as training | |
| **NPV** | | Flu | Flu, RSV | Flu | Flu, RSV |
| PCR | Flu |  | p>0.1 | 0.067 | **0.008** |
|  | Flu, RSV |  |  | **0.01** | **<0.001** |
| PCR+RAT as training | Flu |  |  |  | 0.067 |
|  | Flu, RSV |  |  |  |  |

Supplementary Table 6. Statistical significance of differences in performance scores (DSS-C) for influenza. The Wilcoxon test was used to assess the significance of median differences between groups.

| DSS-P for RSV | | PCR | | PCR+RAT as training | |
| --- | --- | --- | --- | --- | --- |
| **AUC** | | RSV | Flu, RSV | RSV | Flu, RSV |
| PCR | RSV |  | p>0.1 | p>0.1 | p>0.1 |
|  | Flu, RSV |  |  | p>0.1 | p>0.1 |
| PCR+RAT as training | RSV |  |  |  | p>0.1 |
|  | Flu, RSV |  |  |  |  |
| DSS-P for RSV | | PCR | | PCR+RAT as training | |
| **Sensitivity** | | RSV | Flu, RSV | RSV | Flu, RSV |
| PCR | RSV |  | p>0.1 | **0.003** | p>0.1 |
|  | Flu, RSV |  |  | **<0.001** | **0.001** |
| PCR+RAT as training | RSV |  |  |  | **0.016** |
|  | Flu, RSV |  |  |  |  |
| DSS-P for RSV | | PCR | | PCR+RAT as training | |
| **Specificity** | | RSV | Flu, RSV | RSV | Flu, RSV |
| PCR | RSV |  | p>0.1 | 0.054 | p>0.1 |
|  | Flu, RSV |  |  | **<0.001** | **0.007** |
| PCR+RAT as training | RSV |  |  |  | p>0.1 |
|  | Flu, RSV |  |  |  |  |
| DSS-P for RSV | | PCR | | PCR+RAT as training | |
| **PPV** | | RSV | Flu, RSV | RSV | Flu, RSV |
| PCR | RSV |  | p>0.1 | p>0.1 | p>0.1 |
|  | Flu, RSV |  |  | **0.002** | **0.011** |
| PCR+RAT as training | RSV |  |  |  | p>0.1 |
|  | Flu, RSV |  |  |  |  |
| DSS-P for RSV | | PCR | | PCR+RAT as training | |
| **NPV** | | RSV | Flu, RSV | RSV | Flu, RSV |
| PCR | RSV |  | p>0.1 | **0.002** | p>0.1 |
|  | Flu, RSV |  |  | **0.001** | **0.001** |
| PCR+RAT as training | RSV |  |  |  | **0.016** |
|  | Flu, RSV |  |  |  |  |

Supplementary Table 7. Statistical significance of differences in performance scores (DSS-P) for RSV. The Wilcoxon test was used to assess the significance of median differences between groups.

| DSS-C for RSV | | PCR | | PCR+RAT as training | |
| --- | --- | --- | --- | --- | --- |
| **AUC** | | RSV | Flu, RSV | RSV | Flu, RSV |
| PCR | RSV |  | p>0.1 | p>0.1 | p>0.1 |
|  | Flu, RSV |  |  | p>0.1 | 0.071 |
| PCR+RAT as training | RSV |  |  |  | p>0.1 |
|  | Flu, RSV |  |  |  |  |
| DSS-C for RSV | | PCR | | PCR+RAT as training | |
| **Sensitivity** | | RSV | Flu, RSV | RSV | Flu, RSV |
| PCR | RSV |  | p>0.1 | **<0.001** | **0.003** |
|  | Flu, RSV |  |  | **0.004** | **0.001** |
| PCR+RAT as training | RSV |  |  |  | p>0.1 |
|  | Flu, RSV |  |  |  |  |
| DSS-C for RSV | | PCR | | PCR+RAT as training | |
| **Specificity** | | RSV | Flu, RSV | RSV | Flu, RSV |
| PCR | RSV |  | p>0.1 | **<0.001** | **0.001** |
|  | Flu, RSV |  |  | **<0.001** | **<0.001** |
| PCR+RAT as training | RSV |  |  |  | p>0.1 |
|  | Flu, RSV |  |  |  |  |
| DSS-C for RSV | | PCR | | PCR+RAT as training | |
| **PPV** | | RSV | Flu, RSV | RSV | Flu, RSV |
| PCR | RSV |  | p>0.1 | **<0.001** | **<0.001** |
|  | Flu, RSV |  |  | **<0.001** | **<0.001** |
| PCR+RAT as training | RSV |  |  |  | p>0.1 |
|  | Flu, RSV |  |  |  |  |
| DSS-C for RSV | | PCR | | PCR+RAT as training | |
| **NPV** | | RSV | Flu, RSV | RSV | Flu, RSV |
| PCR | RSV |  | p>0.1 | **<0.001** | **0.039** |
|  | Flu, RSV |  |  | **0.007** | **0.002** |
| PCR+RAT as training | RSV |  |  |  | p>0.1 |
|  | Flu, RSV |  |  |  |  |

Supplementary Table 8. Statistical significance of differences in performance scores (DSS-C) for RSV. The Wilcoxon test was used to assess the significance of median differences between groups.

| AUC | IFN | | | RSV | |
| --- | --- | --- | --- | --- | --- |
| Feature set | Only with  top 5 features | With  all features | Only with  top 5 features | | With  all features |
| DSS-P | 0.727 | 0.749 | 0.885 | | 0.907 |
| DSS-C | 0.737 | 0.776 | 0.900 | | 0.924 |

Supplementary Table 9. Performance (AUC) for each pipeline was evaluated using only the top 5 features. The pipeline configuration was selected based on the best-performing combinations shown in Tables 1 and 2.
